# High prevalence of daily and multi-site pain – a cross-sectional population-based study among 3000 Danish adolescents

**DOI:** 10.1186/1471-2431-13-191

**Published:** 2013-11-19

**Authors:** Michael S Rathleff, Ewa M Roos, Jens L Olesen, Sten Rasmussen

**Affiliations:** 1 HEALTH, Aarhus University, Vennelyst Boulevard 9, 8000 Aarhus C, Denmark; 2 Orthopaedic Surgery Research Unit, Aalborg University Hospital, Soendre Skovvej 15, 9000 Aalborg, Denmark; 3 Research Unit for Musculoskeletal Function and Physiotherapy, Institute of Sports Science and Clinical Biomechanics, University of Southern Denmark, Campusvej 55, 5230 Odense M, Denmark; 4 Department of Rheumatology, Aalborg University Hospital, Reberbansgade 15, 9000 Aalborg, Denmark

**Keywords:** Adolescents, Pain, Cohort study, Paediatrics, Pain

## Abstract

**Background:**

Daily pain and multi-site pain are both associated with reduction in work ability and health-related quality of life (HRQoL) among adults. However, no population-based studies have yet investigated the prevalence of daily and multi-site pain among adolescents and how these are associated with respondent characteristics. The purpose of this study was to investigate the prevalence of self-reported daily and multi-site pain among adolescents aged 12–19 years and associations of almost daily pain and multi-site pain with respondent characteristics (sex, age, body mass index, HRQoL and sports participation).

**Methods:**

A population-based cross-sectional study was conducted among 4,007 adolescents aged 12–19 years in Denmark. Adolescents answered an online questionnaire during physical education lessons. The questionnaire contained a mannequin divided into 12 regions on which the respondents indicated their current pain sites and pain frequency (rarely, monthly, weekly, more than once per week, almost daily pain), characteristics, sports participation and HRQoL measured by the EuroQoL 5D. Multivariate regression was used to calculate the odds ratio for the association between almost daily pain, multi-site pain and respondent characteristics.

**Results:**

The response rate was 73.7%. A total of 2,953 adolescents (62% females) answered the questionnaire. 33.3% reported multi-site pain (pain in >1 region) while 19.8% reported almost daily pain. 61% reported current pain in at least one region with knee and back pain being the most common sites. Female sex (OR: 1.35-1.44) and a high level of sports participation (OR: 1.51-2.09) were associated with increased odds of having almost daily pain and multi-site pain. Better EQ-5D score was associated with decreased odds of having almost daily pain or multi-site pain (OR: 0.92-0.94).

**Conclusion:**

In this population-based cohort of school-attending Danish adolescents, nearly two out of three reported current pain and, on average, one out of three reported pain in more than one body region. Female sex, and high level of sports participation were associated with increased odds of having almost daily pain and multi-site pain. The study highlights an important health issue that calls for investigations to improve our understanding of adolescent pain and our capacity to prevent and treat this condition.

## Background

Pain is not an uncommon experience among adolescents [1]. Risk factors for adolescent pain include physical factors such as being involved in work after school hours [2] and psychological factors [3]. The point prevalence of self-reported bodily pain was recently covered in a systematic review by King et al. [4]. They found that prevalence rates ranged substantially between the included studies with back pain ranging from 14–24%, musculoskeletal and lower limb pain from 4–40% and multi-site pain ranging from 4–49%. Pain prevalence rates were 2–3 times higher in females and increased with age for most pain types. Single studies have shown that the respondent characteristics of gender, age and health-related quality of life (HRQoL) are associated with the prevalence of pain [4]. These associations may, however, be confounded by body mass index (BMI) and sports participation, as high sports participation is associated with high prevalence of musculoskeletal pain [5]. Confounding may occur through the positive association between HRQoL and sports participation [6] and through the negative association between BMI and sports participation [7]. Therefore both these variables may be important confounders when investigating the association between respondent characteristics and pain.

Pain in multiple sites of the body in adolescents has been investigated rarely, but among adults, pain in multiple sites of the body has been shown to be more severe and disabling when compared with pain in a single region [8]. A prospective study by Papageorgiou et al. conducted among adults showed that 7 years after the initial self-reported multi-site pain, one third still had multi-site pain [9]. Similarly, pain during adolescence causes a threefold increase in the risk of pain episodes during adulthood [10-12]. These findings are supported by Kamaleri et al. who showed a strong linear association between the number of pain sites and the reduction in overall HRQoL, sleep quality, psychological health [13], and functional ability among adults [14].

The results from research in adolescent pain [4], adult multi-site pain [8], frequency of pain and their association with HRQoL and risk of subsequent pain episodes during adulthood [10,12,15] call for further investigations into adolescent pain. Of particular interest is the prevalence of daily pain and multi-site pain among adolescents, as no population-based study has yet investigated the prevalence of both daily pain and multi-site pain and how these are associated with respondent characteristics.

The purpose of the study is firstly, to determine the prevalence of self-reported almost daily pain and multi-site pain among adolescents aged 12–19 years, and secondly, to determine the association with respondent characteristics such as age, sex, HRQoL, and lifestyle factors such as BMI and participation in sports.

## Methods

### Design

A cross-sectional study was conducted in the autumn of 2011 in a cohort of school children aged 12 to 19 years (Adolescent Pain in Aalborg 2011, the APA2011-cohort). Within this cohort, a randomised study on adolescents with Patellofemoral Pain was nested [16]. This manuscript reports on the cross-sectional data among all participants in the APA2011-cohort. Ethical approval was obtained for the entire study from the Ethics Committee of the North Denmark Region (N-20110020). The Ethics Committee did not require signed consent from each participant, but required that the schools informed the parents about the study and that participation in the study was voluntary. The reporting of the study complies with the ‘Strengthening the Reporting of Observational studies in Epidemiology’ (STROBE) statement [17].

### Study population

In the area where the study was conducted, there are 38 schools and four upper secondary schools. All 42 schools were contacted, of which eight schools and four upper secondary schools agreed to participate. The schools were all government-funded, the most common type in Denmark. All schools have students from urban, suburban and rural communities with low, middle and high socioeconomic status. No individual specific socioeconomic data were obtained. The schools were not informed of the specific content of the questionnaire before agreeing to participate.

### Procedure

All adolescents aged 12–19 years were invited to answer the online questionnaire during their physical education lessons. Before the data collection, the primary author visited all schools that agreed to participate and informed them about the content of the questionnaire and the purpose of the study. More specifically, a written leaflet was distributed among adolescents with the title ‘Please help answer a questionnaire for a scientific study on physical activity, quality of life and pain’. The leaflet contained information that the study was being conducted by the Orthopaedic Surgery Research Unit at the local hospital together with the Graduate School of Health Sciences at Aarhus University. In addition, a detailed description in the leaflet contained information of interest on the association between physical activity, quality of life and musculoskeletal pain in general, but especially knee pain.

The physical education teachers were instructed to send an email to all adolescents containing a hyperlink to the online questionnaire, inviting adolescents to answer the questionnaire on their own personal computer during the first part of their physical education lessons. This approach ensured confidentiality from physical education teachers while responding to the questionnaire. However, during the data collection phase, not all schools were able to allocate the required time during the physical education lessons, which resulted in some adolescents answering the questionnaire during their lunch break. Adolescents exempt from physical education at that time because of pain or similar conditions still participated in the first part of the physical education lessons.

### Questionnaire

The online questionnaire was designed in collaboration with UNI-C, which is a publicly-funded agency working under The Danish Ministry of Children and Education. The questionnaire was a web-based questionnaire designed to run within all available Internet browsers. To maximise comprehension of the questionnaire by adolescents, the questionnaire was piloted in a sample of 10 adolescents aged 12–19 years.

The online questionnaire contained demographic questions on age, sex, height, weight and the school they attended. After answering these questions, the adolescents were presented with a pain mannequin and they were instructed to mark the regions where they currently experienced pain [18]. The mannequin was shown included both a frontal and posterior view of the human body, with the regions of the body written in letters beside the corresponding body segment. Adolescents had the option of clicking on the name of the region, or pressing the specific region of the body where they were experiencing pain. After they had selected a region, it became grey so they could see which regions of the body they had selected. Afterwards, they were asked separately about the pain frequency in the regions they had selected. The frequency of pain was chosen as a quick and easily comprehensible measure of pain. Based on previous studies using the same approach, the pain frequency was divided into the following five categories: rarely; monthly; weekly; more than once per week; almost daily [19,20].

After the pain mannequin questions, the adolescents were asked if they participated in sports besides the mandatory physical education classes during school hours. If they participated in sports, they were asked about which types and how many times they participated each week.

The last page of the questionnaire contained the EuroQoL 5D (EQ-5D) which measures HRQoL [21]. The questionnaire was available in two versions depending on the age of the participating adolescent. The questionnaire for the adolescents aged 15–19 contained the adult version of EQ-5D [21]. The EQ-5D is a five-dimensional health state classification. The five dimensions are mobility, self-care, usual activities, pain/discomfort and anxiety/depression. The five dimensions are each assessed by a single question on a three-point ordinal scale (no problems, some problems, extreme problems). The questionnaire for the adolescents aged 12–14 years contained the youth version of the EQ-5D-Y with the wording suggested by Burström et al. [22]. Like the adult version, it consists of a descriptive system that comprises five items. The wording is slightly different to ensure a better comprehension by younger adolescents. Mobility was replaced with “walking about”, self-care “looking after myself”, usual activities “doing usual activities”, pain and discomfort “having pain or discomfort”, and anxiety and depression was replaced with “feeling worried, sad or unhappy”. In both the EQ-5D and EQ-5D-Y a ‘health state’ is defined by selecting one level from each of the five dimensions. A total of 243 health states are thus defined. The Danish time-trade-off (TTO) scoring algorithm was then used to weight each respondent’s profile data to derive a single EQ-5D index score [23]. The EQ-5D index score can be regarded as a continuous outcome scored on a -0.59 to 1.00 scale, with 1.00 indicating ‘full health’, 0 representing dead while negative scores represent health states valued as worse than dead [21]. Depending on the disease condition, the minimal important difference is 0.074 with a range from -0.011 to 0.140 [24].

### Definition of single region and multi-site pain

Region-specific pain was defined as pain in only one region of the body, while multi-site pain was defined as pain in at least two of the 12 predefined regions. The predefined region-specific pain sites were: head, shoulder, back, elbow, hip/groin, thigh, knee, shin, feet, chest, stomach, lower arm and hand [18].

### Data analysis

The prevalence of pain was calculated according to age and sex and presented descriptively. Between schools, comparisons were made using the prevalence rates together with the 95% confidence interval to look for overlapping confidence intervals.

Similar to previous studies within the same area, univariate and multivariate regression analyses were used to explore the associations between respondent characteristics and multi-site and almost daily pain [14,25,26]. The following variables were investigated: age, sex, HRQoL, the number of sessions of sport participation per week, and BMI (divided into quartiles). If the p-values were ≤0.15 in the univariate analysis, the variable was included in a multivariate analysis with robust variance estimates that adjust for within-cluster correlations within schools. In the multivariate analysis, a variable with a p-value >0.05 was only retained in the model if its exclusion caused a change of more than 10% in the odds ratio of the other included variables. Previous studies have found a higher prevalence of self-reported pain among females and therefore we considered sex a potential effect modifier. However interaction between sex and explanatory variables were tested and no significant interaction was found. Stata (Stata Corp, Texas, USA. Version 11) was used for all statistical analyses.

## Results

### Demographics

4.007 adolescents aged 12–19 years were approached and 2,953 (73.7%) answered the questionnaire correctly and were included in the data analysis. 799 did not participate, while 255 only partially completed the questionnaire and were excluded from the analysis, (Table 1).

**Table 1:** Cohort characteristics for male and females across all 12 schools

	**Male (n = 1126)**	**Female (n = 1827)**
Age (years), median (IQR)	17 (15–18)	17 (16–18)
Height (cm), mean (SD)	177.8 (10.1)	167.2 (6.9)
Weight (kg), mean (SD)	67.2 (13.8)	58.8 (11.2)
BMI (kg/m^2^), median (IQR)		
14.0-18.9	256 (22.7%)	483 (26.5%)
18.9-20.7	265 (23.5%)	475 (26.0%)
20.7-22.6	304 (27.0%)	433 (23.7%)
22.6-40.3	301 (26.8%)	435 (23.8%)
Sports participation per week (IQR)	3 (0–4)	2 (0–3)

IQR = Interquartile Range.

In primary and lower secondary schools, the proportion of females varied from 43% to 67%, which reflects the true distribution of 49% females. In upper secondary schools, the percentage of females varied between 56% and 68% across the different schools, which reflects the true distribution of 61% females. Non-responders were equally divided across all age groups (data not shown). No significant difference was found between schools and therefore, results are presented for the total study population.

#### Almost daily pain and multi-site pain

61% of the adolescents reported pain in at least one region, while almost 20% reported almost daily pain, (Table 2). 984 adolescents (33.3% of the cohort) experienced multi-site pain, i.e. pain from more than one of the 12 pre-specified regions, (Table 3). Almost daily pain and multi-site pain were more common among females and the prevalence increased with age.

**Table 2:** Prevalence of almost daily pain in the full cohort and by age and gender

	All (% of all adolescents in that age group (95% CI*))	Male (% of all males in that age group (95% CI*))	Female (% of all females in that age group (95% CI*))
12-19 years, n = 2953	19.8 (18.4-21.2)	13.3 (11.3-15.3)	23.8 (21.8-25.7)
12 years, n = 123	10.6 (5.1-16.0)	7.8 (0.3-15.3)	12.5 (4.8-20.2)
13 years, n = 186	20.4 (14.6-26.2)	15.4 (7.3-23.4)	24.1 (16.0-32.2)
14 years, n = 241	19.1 (14.1-24.1)	13.0 (6.6-19.3)	24.1 (16.8-31.4)
15 years, n = 248	18.5 (13.7-20.1)	16.1 (9.4-22.8)	20.8 (13.8-27.8)
16 years, n = 532	16.9 (13.7-20.1)	12.6 (7.8-17.4)	19.2 (15.1-23.3)
17 years, n = 753	23.6 (20.5-26.6)	15.4 (10.9-20.0)	27.5 (23.6-31.4)
18 years, n = 630	19.8 (16.7-23.0)	12.0 (7.8-16.1)	24.5 (20.3-28.7)
19 years, n = 240	20.8 (15.7-26.0)	11.3 (5.3-17.4)	28.4 (20.7-36.0)

*95% Confidence intervals.

**Table 3:** Percentage of males and females having multi-site pain

	**All**	**Males (% in that age group)**	**Females (% in that age group)**
12-19 years, n = 2953	33.3 (31.6-35.0)	25.0 (22.4-27.5)	38.5 (36.2-40.7)
12 years, n = 123	21.1 (13.9-28.4)	19.6 (8.6-30.6)	22.2 (12.5-31.9)
13 years, n = 186	30.1- (23.5-36.7)	29.5 (19.3-39.7)	30.6 (21.8-39.3)
14 years, n = 241	30.3 (24.5-36.1)	25.0 (16.8-33.2)	34.6 (26.5-42.7)
15 years, n = 248	29.0 (23.4-34.7)	18.6 (11.6-25.7)	38.5 (30.1-46.9)
16 years, n = 532	32.9 (28.9-36.9)	24.0 (17.8-30.3)	37.5 (32.4-42.6)
17 years, n = 753	36.0 (32.5-39.4)	24.4 (19.0-29.8)	41.6 (37.3-45.9)
18 years, n = 630	34.9 (31.2-38.6)	28.2 (22.4-34.0)	38.9 (34.1-43.7)
19 years, n = 240	38.3 (32.2-44.5)	27.4 (18.8-35.9)	47.0 (38.5-55.5)

#### Prevalence and frequency of self-reported region-specific pain

**Figure 1:**
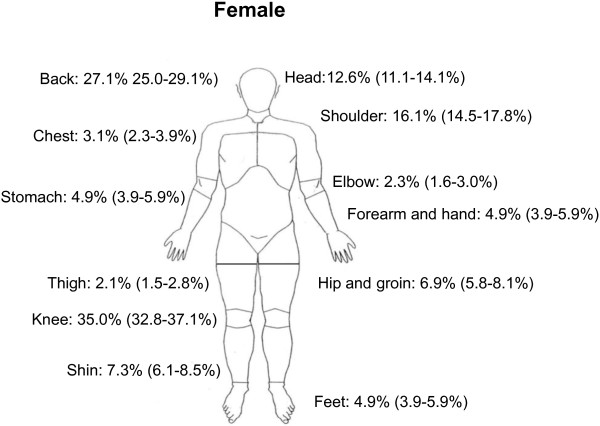
Prevalence rates of region-specific pain for females (n = 1828).

**Figure 2:**
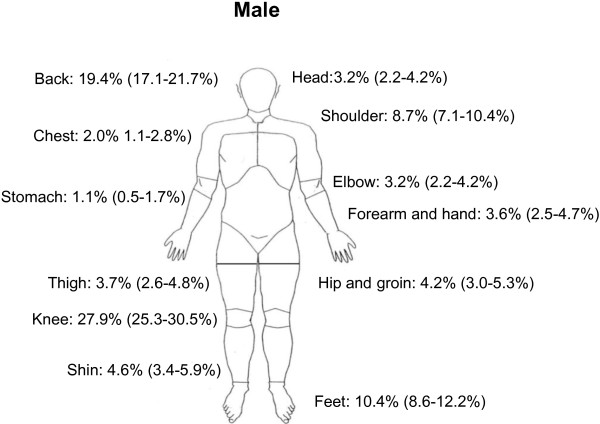
Prevalence rates of region-specific pain for males (n = 1125).

Knee pain was the most prevalent self-reported region of pain (32.3%), (Table 4, Figures 1 and 2). The prevalence of knee pain was higher for females (35.0%) compared with males (27.9%). Back pain was the second most commonly reported region of pain. Nearly 25% experienced almost daily pain from the knees or back. Females generally reported a higher prevalence of pain, especially in the head, shoulder, back and knee.

**Table 4:** Prevalence and frequency of self-reported region-specific pain

		**Frequency of pain (% among adolescents with pain)**				
**Body region**	**% of all adolescents reporting region-specific pain with any frequency**	**Almost daily**	**More than once per week**	**Weekly**	**Monthly**	**Rarely**
Knee, n = 953	32.3 (30.6-34.0)	25.1% 22.3-27.8	17.9% 15.4-20.3	24.9% 22.1-27.6	25.8% 23.0-28.6	6.3% 4.8-7.9
Back, n = 713	24.1 (22.6-25.7)	23.5% 20.3-26.6	21.1% 18.1-24.1	24.8% 21.6-27.9	25.3% 22.1-28.5	5.4% 3.7-7.0
Shoulder, n = 393	13.3 (12.1-14.5)	27.4% 23.0-31.9	21.0% 16.9-25.1	22.3% 18.2-26.5	23.3% 19.1-27.5	5.9% 3.5-8.2
Foot, n = 341	11.5 (10.4-12.7)	24.8% 20.1-29.4	18.5% 14.3-22.7	23.9% 19.3-28.5	23.9% 19.3-28.5	9.0% 5.9-12.0
Head, n = 266	9.0 (8.0-10.0)	23.1% 18.0-28.2	24.2% 19.0-29.4	22.7% 21.9-32.7	22.7% 17.6-27.8	2.7% 0.7-4.6
Shin, n = 185	6.2 (5.4-7.1)	14.3% 9.2-19.4	18.7% 13.0-24.4	27.5% 20.9-34.0	29.7% 23.0-36.4	9.9% 5.5-14.3
Hip/groin, n = 174	5.9 (5.0-6.7)	19.4% 13.4-25.4	21.2% 15.0-27.4	20.0% 13.9-26.1	32.4% 25.2-39.5	7.1% 3.2-10.9
Forearm/hand, n = 130	4.4 (3.7-5.1)	25.6% 18.0-33.2	18.6% 11.8-25.4	23.3% 15.9-30.6	20.2% 13.1-27.2	12.4% 6.6-18.2
Stomach, n = 101	3.4 (2.8-4.1)	13.3% 6.4-20.1	13.3% 6.4-20.1	29.6% 20.4-38.8	38.8% 29.0-48.6	5.1% 0.7-9.5
Thigh, n = 81	2.7 (2.2-3.3)	20.5% 11.3-29.7	19.2% 10.3-28.2	21.8% 12.4-31.2	24.4% 14.6-34.1	14.1% 6.2-22.0
Elbow, n = 78	2.6 (2.1-3.2)	29.9% 19.4-40.3	13.0% 5.3-20.7	16.9% 8.3-25.4	28.6% 18.3-38.9	11.7% 4.3-19.0
Chest, n = 78	2.6 (2.1-3.2)	9.0% 2.5-15.5	19.2% 10.3-28.2	28.2% 18.0-38.4	35.9% 25.0-46.8	7.7% 1.6-13.7

#### Associations between respondent characteristics and odds of having almost daily pain and multi-site pain

The multivariate logistic regression analysis showed that female sex, and worse HRQoL were associated with increased odds of having almost daily pain and multi-site pain (Tables 5 and 6). Participation in sports more than six times per week was associated with increased odds of having almost daily pain, while participating in sports more than three times per week was associated with increased odds of multi-site pain. Further, a BMI >22.6 was associated with increased odds of having multi-site pain, but not almost daily pain. There was a positive association between almost daily pain and multi-site pain meaning reporting daily pain increased the odds of reporting multi-site pain and vice-versa. No other associations of respondent characteristics or lifestyle factors with reported pain were found.

**Table 5:** Odds for having almost daily pain

**Odds for having almost daily pain**	**Crude coef.**	**p-value coef**	**Adjusted coef**	**p-value adj coef**	**[95% CI] Adj OR**
Older age	1.06	0.08	0.97	0.34	0.91-1.03
Female gender	2.03	<0.0001	1.35	0.04	1.02-1.68
BMI (compared to quartile 0-25%, 14.0-18.9)					
BMI quartile 25-50% (18.9-20.7)	1.10	0.32	1.08	0.63	0.78-1.51
BMI quartile 50-75% (20.7-22.6)	1.23	0.11	1.12	0.33	0.89-1.43
BMI quartile 75-100% (22.6-40.3)	1.40	0.02	1.07	0.64	0.80-1.43
EQ-5D index score (per 0.1 point increase)	0.91	<0.0001	0.92	<0.0001	0.91-0.93
Multi-site pain	6.79	<0.0001	3.14	<0.0001	2.35-4.19
Sports participation per week (compared with 0 times per week)					
1	1.08	0.71	1.18	0.61	0.63-2.23
2	0.91	0.53	0.87	0.43	0.61-1.24
3	0.88	0.58	0.94	0.84	0.52-1.71
4	1.13	0.48	1.19	0.38	0.80-1.78
5	1.05	0.82	1.34	0.26	0.81-2.22
6	1.28	0.10	1.46	0.16	0.86-2.48
7	1.80	0.002	2.09	0.003	1.28-3.39

Univariate and multivariate logistic regression analysis. Crude coefficient shows the result from the univariate logistic regression analysis between almost daily pain and each of the six explanatory variables. Adjusted coefficients show the results from the multivariate analysis.

**Table 6:** Odds for having multi-site pain

**Odds for having multi-site pain**	**Crude coef.**	**p-value coef**	**Adjusted coef**	**p-value adj coef**	**[95% CI] Adj OR**
Older age	1.09	0.005	1.02	0.60	0.96- 1.09
Female gender	1.88	<0.0001	1.44	0.01	1.09-1.90
BMI (compared to quartile 0-25%, 14.0-18.9)					
BMI quartile 25-50% (18.9-20.7)	1.26	0.004	1.19	0.29	0.87- 1.63
BMI quartile 50-75% (20.7-22.6)	1.37	0.03	1.28	0.09	0.97 -1.69
BMI quartile 75-100% (22.6-40.3)	1.60	<0.0001	1.35	0.04	1.00- 1.80
EQ-5D index score (per 0.1 point increase)	0.93	<0.0001	0.94	<0.0001	0.94- 0.95
Almost daily pain	6.79	<0.0001	2.85	<0.0001	2.18-3.72
Sports participation per week (compared with 0 times per week)					
1	1.25	0.24	1.36	0.26	0.80-2.31
2	1.31	0.004	1.38	0.05	1.00-1.92
3	1.39	0.08	1.63	0.01	1.11-2.38
4	1.37	0.004	1.51	0.01	1.19-1.92
5	1.38	0.005	1.80	0.001	1.27-2.55
6	1.46	0.04	1.65	0.09	0.92-2.97
7 or more	1.66	<0.0001	1.71	0.005	1.18-2.50

Univariate and multivariate logistic regression analysis. Crude coefficient shows the result from the univariate logistic regression analysis between multi-site pain and each of the six explanatory variables. Adjusted coefficients show the results from the multivariate analysis.

#### Health-related quality of life in adolescents

**Figure 3:**
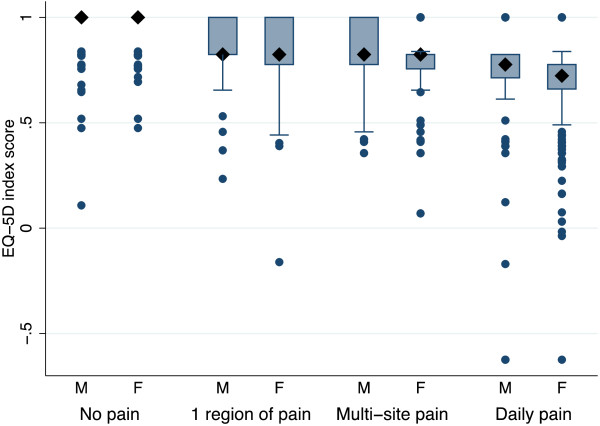
**Box and whisker plot for EQ-5D index score.** The EQ-5D index score is divided into adolescents with no pain, one region of pain, multi-site pain, and daily pain. The boxes are bordered at the 25th and 75th percentile (the interquartile range (IQR)) and the diamonds represent the median EQ-5D index scores. The whiskers display the boundary of 1.5 times the interquartile range above and below the median. Outliers beyond the whiskers are shown as individual circles.

Males and females who reported one region of pain, almost daily pain or multi-site pain had worse HRQoL than those without pain (p < 0.0001) (Figure 3). Males and females with almost daily pain had a significantly lower HRQoL than those who reported pain from one region or multi-site pain (p < 0.03).

## Discussion

This school-based survey adds important knowledge about adolescent self-reported pain and shows that pain is already very common during adolescence. The study showed that 60% of this population-based cohort of Danish school-attending adolescents reported current bodily pain. Twenty per cent of all adolescents reported almost daily pain and almost 35% reported multi-site pain. The knee and back were the two most prevalent regions of self-reported pain. Female sex, and high level of sports participation were associated with increased odds of having multi-site and almost daily pain while better self-reported HRQoL decreased the odds of almost daily pain and multi-site pain.

### Methodological considerations

The study was a population-based survey with a large sample size from a single municipality in Denmark consisting of a mix of adolescents from urban, suburban and rural communities. This is the first study in Denmark investigating almost daily pain and multi-site pain among adolescents aged 12–19 years. However, generalisability of our results may be limited due to our response rate of 73.7% and the low participation rate of schools. However schools and students were not aware of the specific content of the questionnaire before accepting to participate. Therefore, we expect a low risk of bias due to school non-participation. The leaflets distributed to schools contained information explaining that the Orthopaedic Surgery Research Unit was responsible for the survey and that we were interested in physical activity, quality of life and pain. The inside of the leaflet contained information stating that knee pain was of particular interest. The reason for this was that we were interested in both the prevalence of pain and recruitment of adolescents with knee pain for a randomised study as stated in this study protocol [16]. The leaflet could have biased the prevalence of knee pain but it was unlikely to have influenced the prevalence of almost daily pain and multi-site pain nor the associations between these and the respondent characteristics.

We have no data on self-reported pain experienced by non-responders; however, there was no difference in respondent characteristics such as age and sex. Studies suggest that responders tend to have better health than dropouts, which would cause an under-reporting of self-reported pain [27,28]. The opposite may also hold true, as individuals experiencing pain may be more interested in the study and therefore may decide to participate [29]. A selection bias may influence the prevalence of almost daily pain and multi-site pain but it is unlikely that the association between respondent characteristics and almost daily pain and multi-site pain would be affected in such a way that it would alter our conclusions.

A further limitation of our study was that we did not include a direct measure of pain intensity or duration of pain. Our pilot testing revealed that we needed to keep the questionnaire short and simple without the need to read a long introduction before answering the questionnaire. The adolescents between 15 and 19 years of age in upper secondary school, in particular, reported that the questionnaire had to be short and simple if they were to answer it. This significant limitation may question the relevance of the pain experience, and needs to be considered when interpreting and generalising our results. Since information about pain duration was not collected, reported pain may include both short-term and long-standing pain. This population-based sample includes highly heterogeneous pain complaints and may include both adolescents with multiple distinct regions of pain and adolescents with more strictly defined chronic widespread pain [30]. The questionnaire did not include questions about menstrual cycle, which may partly explain the increased prevalence of self-reported hip, groin or stomach pain among females.

As the work on EQ-5D TTO scores specific to the EQ-5D-Y version is on-going, but unpublished, we used the TTO scores from the adult version of the EQ-5D to calculate the EQ-5D-Y index scores. This approach had been used earlier but it is important to state that no youth-specific TTO scores exist [31,32]. The lack of children-specific utility weights in the calculation of TTO scores may decrease the external validity of the TTO scores to other countries because of different health valuations.

### Prevalence of almost daily pain and multi-site pain

The questionnaire included a question on the frequency of pain, which allowed us to investigate the prevalence of almost daily pain. The results showed that almost 20% of the adolescents experienced almost daily pain, with females having approximately 50% higher prevalence than males (19.8% vs 13.3%). The most common pain sites were the knee and back. Back pain prevalence of 24% is similar to that found in a previous Danish study on back pain among adolescents [33] and is almost identical to that reported by King et al. [4] where the prevalence of back pain ranged from 14-24% with a median of 21%. The prevalence of knee pain is similar to that reported in a recent study on knee pain among adolescents aged 16–18 years, where the prevalence was 25.0% [34]. Interestingly, knee and back pain were also among the most common types of musculoskeletal pain in a recent population-based study among adult Danes [35].

The high prevalence of multi-site pain found in our study is in line with previous studies on adolescents [1,19,26]. Among Swedish children, the co-occurrence of pain in the head, stomach, and back was common and half the adolescents with pain symptoms reported multi-site pain at least monthly [36]. Similarly, 27% of Dutch adolescents reported multi-site pain, with females having twice as high a prevalence as males [1]. Almost 20% of the adolescents in our population reported almost daily pain, which shows that the prevalence of almost daily pain among adolescents closely resembles that found among adults [37,38].

### The association between respondent characteristics and the odds of having multi-site or almost daily pain

The association between almost daily pain, multi-site pain, and HRQoL indicates that pain has negative physical, mental, and social consequences [39]. Factors such as pain intensity and pain frequency, duration of pain, and higher expectations of pain determine the extent of reduction in quality of life among individuals with musculoskeletal pain [40,41]. We did not include duration of pain or expectation of pain but we did find a reduction in HRQoL among those with almost daily pain and multi-site pain, which is similar to previous studies on adolescents and young adults [41,42].

Both before and after adjusting for HRQoL, BMI, age and sports participation, the results showed that females were almost twice as likely to report almost daily pain or multi-site pain than males. Increased prevalence among females was also one of the consistent findings across studies in the review on adolescent pain done by King et al. [4]. It is unknown if the increased prevalence among females is the result of a physiologically altered pain perception or associated with biopsychosocial factors such as pain catastrophisation, anxiety, and depression which were not specifically accounted for in the current study. Similar to King et al., we found an increasing prevalence of pain with increasing age [4]. We found a doubling in the prevalence of almost daily pain from age 12 to 13 years among females, which may be associated with the mean age of menarche at age 13 in Denmark [43]. Since this doubling was found at all pain sites, also remote to the abdomen, a hormonal link is possible [44]. Linking these findings to a prevalence of almost daily pain of 23.7% and multi-site pain of 38.5% among female adolescents indicates an important issue that needs further investigation into potential treatment and underlying causes.

A high level of sports participation was associated with increased risk of almost daily pain and multi-site pain. This finding is in accordance with a previous population-based study showing that adolescents with a high level of sports participation have higher prevalence of self-reported pain [5]. As suggested by Kujala et al., physical activity has important health benefits but there could be an upper limit to the amount of exercise recommended [5]. Additionally this has implications for future studies investigating adolescent pain, as sports participation may be an important confounder and may help distinguish between sports-related pain and chronic pain conditions [5].

### Implications

Our findings may have future societal consequences, as adolescents with pain are more likely to report pain during early adulthood and later in life [12]. Some authors even speculate that chronic pain conditions in adults may start during adolescence [45]. To improve HRQoL during adolescence and prevent an increase in future chronic pain conditions, we need to target the large group of adolescents with almost daily pain and multi-site pain. Early intervention may in some cases or conditions improve the chance of becoming pain-free later [46,47].

## Conclusion

More than 20% of school-attending adolescents from this Danish population-based cohort reported almost daily pain while almost 35% reported multi-site pain. Almost daily pain and multi-site pain were more common in females and in the older age group and were associated with worse health-related quality of life. Our findings suggest that pain is already common during adolescence and that to improve quality of life during adolescence and reduce chronic pain during adulthood, we should increase our focus on adolescents experiencing musculoskeletal and other pain.

## Competing interests

The authors declare that they have no competing interests.

## Authors’ contributions

All authors made substantial scientific contributions to the design of the trial. MSR wrote the first draft for this manuscript. EMR, JLO and SR all made valuable scientific additions to the draft. All authors read and approved the final manuscript.

## Pre-publication history

The pre-publication history for this paper can be accessed here:

http://www.biomedcentral.com/1471-2431/13/191/prepub
